# Long QT syndrome in chromosome 7q35q36.3 deletion involving *KCNH2* gene: Warning for chlorpheniramine prescription

**DOI:** 10.1002/mgg3.855

**Published:** 2019-07-25

**Authors:** Giuseppe Di Stolfo, Maria Accadia, Sandra Mastroianno, Maria P. Leone, Orazio Palumbo, Pietro Palumbo, Domenico Potenza, Pasquale Maccarone, Michele Sacco, Aldo Russo, Massimo Carella

**Affiliations:** ^1^ Cardiovascular Department Fondazione IRCCS Casa Sollievo della Sofferenza San Giovanni Rotondo Foggia Italy; ^2^ Medical Genetics Service Hospital “Cardinale G. Panico” Tricase Lecce Italy; ^3^ Division of Medical Genetics Fondazione IRCCS Casa Sollievo della Sofferenza San Giovanni Rotondo Foggia Italy; ^4^ Paediatric Unit Fondazione IRCCS Casa Sollievo della Sofferenza San Giovanni Rotondo Foggia Italy

**Keywords:** chlorpheniramine, chromosome 7q35q36.3 deletion, long QT syndrome, syncope

## Abstract

**Background:**

The deletion of the distal 7q region is a rare chromosomal syndrome characterized by wide phenotypic manifestations including growth and psychomotor delay, facial dysmorphisms, and genitourinary malformations.

**Methods:**

We describe a 6‐year‐old child with a 12‐Mb deletion of the region 7q35q36.3.

**Results:**

Among the deleted genes, two genes have cardiac implications: *PRKAG2* (OMIM #602743), associated with hypertrophic cardiomyopathy, cardiac conduction disease, and sudden death, and *KCNH2* (OMIM #152427), coding for a cardiac potassium channel involved in long QT syndrome, unmasked by the chlorpheniramine treatment. At same time, the SHH gene (OMIM #600725), encoding sonic hedgehog, a secreted protein that is involved in the embryonic development, is deleted.

**Conclusion:**

Our report underlines potential cardiac complications linked to the common pharmacological treatment in this rare multiorgan and proteiform disease.

## 1 INTRODUCTION

The deletion of the distal 7q region is a rare chromosomal syndrome characterized by wide phenotypic manifestations including growth and psychomotor delay, facial dysmorphisms, and genitourinary malformations; more severe phenotypes, such as sacral agenesis and holoprosencephaly (HPE) may occur (Harris et al., 1977; Vance et al., 1998; Wang et al., 1999). We describe a 6‐year‐old child with a 12‐Mb deletion of the region 7q35q36.3. Among the deleted genes, two genes have cardiac implications: *KCNH2*, coding for a cardiac potassium channel involved in long QT syndrome (LQTS) (Perrin, Subbiah, Vandenberg, & Hill, 2008); and *PRKAG2*, associated with hypertrophic cardiomyopathy, cardiac conduction disease, and sudden death (Porto et al., 2016). Our report underlines the relationship among potential cardiac complications and rare multiorgan and proteiform disease, with pharmacological implications in the daily management.

## 2 CLINICAL REPORT

**Figure 1:**
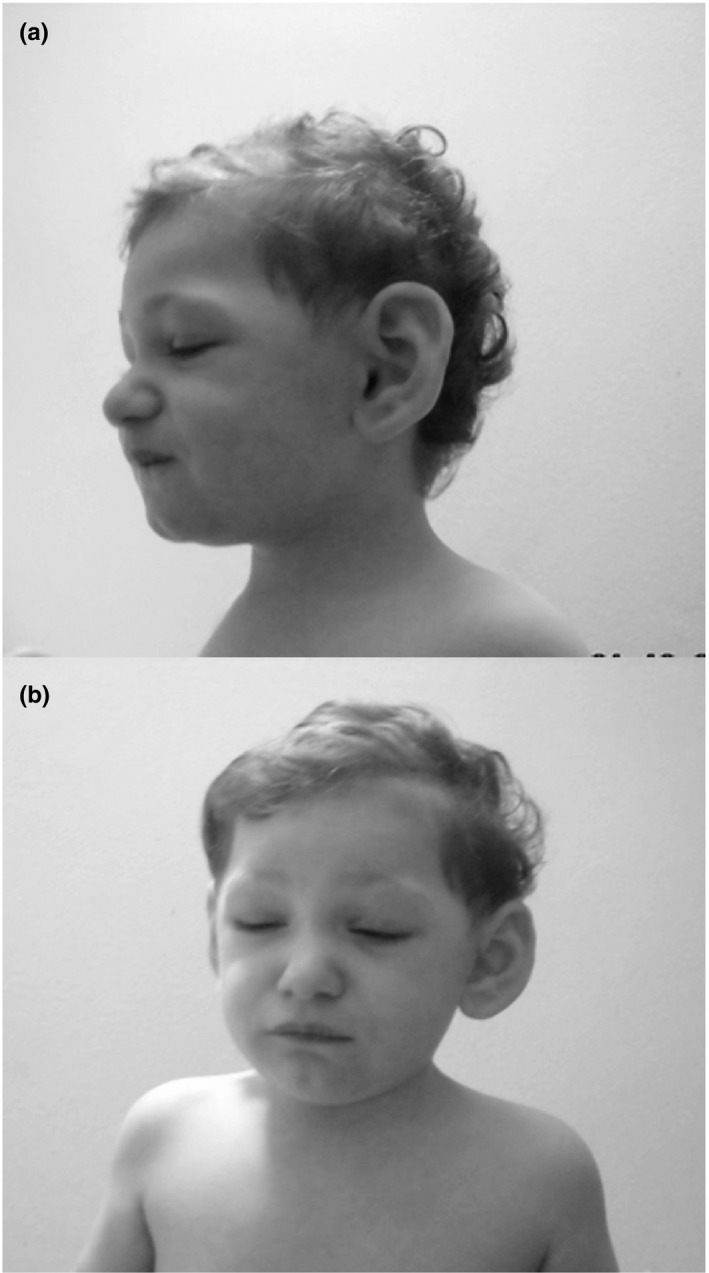
Clinical features (a and b show frontal and lateral view)

A 6‐year‐old child was admitted to our Pediatric Department after a prolonged self‐limited episode of loss of consciousness while he was at school. The teacher described a 30‐min loss of consciousness, pallor, and hypotonia with generalized cyanosis compatible with an *ALTE* (*Apparent Life‐Threatening Event*) (Piumelli et al., 2017). The child was already known to our hospital for a previous diagnosis of 12‐Mb de novo deletion of the region 7q35q36.3. His clinical picture was characterized by growth and psychomotor delay, hypotonia, microcephaly (occipitofrontal head circumference 42.6 cm, below −3 *SD* for age and sex), and craniofacial dysmorphic features (bitemporal narrowing, prominent supraorbital ridges, eyelid ptosis, deep‐set eyes, bulbous nasal tip, microretrognathia, high palate, and large protruding ears) (Figure 1a,b). An echocardiogram performed in the first month of life showed ventricular septal defect, common in this chromosome deletion (Tiller et al., 1988), and self‐resolved at subsequent control. At the age of 4 years, he underwent urologic surgery for curved penis and hypospadias. A recent brain MRI did not show significant abnormalities. The presence of high palate resulted in feeding problem and rhinorrhea, with recurrent infections of the upper respiratory airways characterized by glue ear and rhinogenous deafness. These complications were often treated by steroids and antihistamines.

**Figure 2:**
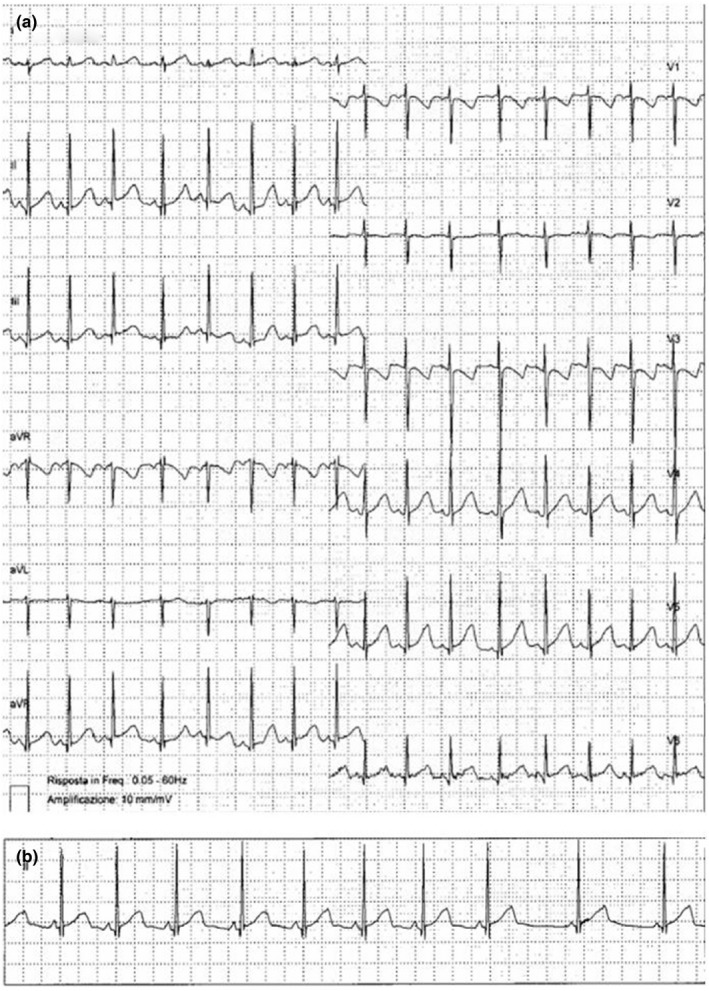
(a) Long QT interval after clorpheniramine consumption. (b) Evidence of short PR interval and normal QT interval

At the time of admission, vital signs were normal and the ECG showed sinus rhythm and normal ventricular repolarization. The patient underwent complete evaluation; in particular, the echocardiogram was normal and subsequent EEGs did not reveal epileptiform anomalies. A 24‐hr Holter ECG recording showed repetitive QTc prolongation (QTc 520 ms, Figure 2a), concurrent with daily consumption of syrup containing acetaminophen and chlorpheniramine; another relevant ECG features was the short PR interval, up to 80 ms (Figure 2b).

The 7q35q36.3 region contains the *KCNH2* gene, coding for the human Ether‐à‐go‐go‐related gene (hERG) potassium channel involved in LQTS and chlorpheniramine represents a plausible hERG potassium blocker in an already compromised receptor malfunction. Since the strict correlation between syrup consumption and QT prolongation was manifest, the parents were advised to avoid a list of proarrhythmic drugs to the patient affected by LQTS ([Link]). Moreover, the deletion includes the *PRKAG2* gene, implicated in a nonsarcomeric form of hypertrophic cardiomyopathy, associated with accessory pathway, short PR, and late evidence of supraventricular and ventricular tachycardia, complete heart block, and sudden death. The suspect for a seizure episode remained as the hypothesis of a prolonged self‐terminated ventricular arrhythmia was not documented and the need for a secondary prevention ICD implantation did not fulfill current guidelines. Therefore, the patient was referred to a tertiary cardiology pediatric center for loop recorder implantation, to better assess future hypotonic episode, and to avoid the dangerous arrhythmic event.

## 3 MATERIALS AND METHODS

SNP array‐based copy number variations (CNVs) analysis was performed on genomic DNA extracted from peripheral blood lymphocytes of the patient and his parents using the CytoScan HD Array (Affymetrix) as previously described (Palumbo et al., 2014). Data analysis was performed using the Chromosome Analysis Suite software version 3.1 (Affymetrix). A CNV was validated if at least 25 contiguous probes showed an abnormal log2 ratio.

## 4 ETHICAL COMPLIANCE

Informed consent, approved by the Fondazione IRCCS Casa Sollievo della Sofferenza Ethical Committee, was obtained from both parents for the genetic analysis, case history, and picture publication.

## 5 RESULTS

An interstitial 12‐Mb deletion of the chromosome 7q35‐36 was observed, a mapping between linear sequence locations 143,873,921 and 155,888,203, according to the GRCh37/hg19 build of the human genome. No other clinically significant copy number changes were detected. Parental analysis using the same platform yielded normal results and the deletion identified in the patient was determined to be de novo. The molecular karyotype of the patient, according to the ISCN 2016, was: arr[GRCh37] 7q35q36.3 (143873377x2,143873921‐155888203x1, 155888261x2)dn.

The deleted region resulted in the loss of one copy of 131 genes, including *SHH*, *KCNH2*, and *PRKAG2*.

## 6 DISCUSSION

Complex syndromes originating from the deletion of the region concerning multiple genes are characterized by multisystemic involvement. Dysmorphisms and systemic malformations generally arise medical attention at the beginning, while further associated manifestations need a methodological approach concerning both genetic analysis and clinical evaluation.

In common practice, upper respiratory way complications secondary to palate involvement often cause a clinician's Pavlovian reflex leading to corticosteroid and antihistamines prescription. In this case, chlorpheniramine consumption was clearly associated with QT prolongation.

According to the DECIPHER database, the haploinsufficiency index for *KCNH2* is 8.86%, indicating that not only the point mutations but also its deletion could lead to deleterious effect (Huang, Lee, Marcotte, & Hurles, 2010). Three other patients harboring the deletion of distal 7q encompassing *KCNH2* were found to have a LQTS (Bisgaard, Rackauskaite, Thelle, Kirchhoff, & Bryndorf, 2006; Caselli et al., 2008).

Previous literature suggests that chlorpheniramine, an H1 antihistamine, is a blocker of the hERG channels, providing a molecular mechanism for the drug‐induced arrhythmogenic side effects (Hong & Jo, 2009). Indeed, the hERG potassium ion channel plays a key role in cardiotoxicity and is therefore a key target as a part of preclinical drug discovery toxicity screening (Shen, Su, Esposito, Hopfinger, & Tseng, 2011; Sun, Xia, Austin, & Huang, 2012). Altogether, these observations underline a clear role of the pharmacogenomic integrated approach in daily practice toward rare complex disease, leading to a desirable personalized medicine (Salari, Watkins, & Ashley, 2012).

Nevertheless, the screening approach by means of electrocardiography should play a key role in the management of multifaceted genetic disorder to avoid arrhythmic complication, in both single case treatment and global awareness by building a gene‐related International Registry (Narayanan & Chugh, 2015).

The phenotypic involvement of *PRKAG2* expression may be realized in the previous ventricular septal defect self‐resolved and in the presence of short PR (up to 80 ms). PRKAG2 syndrome is characterized by ventricular preexcitation, supraventricular arrhythmias, and cardiac hypertrophy, often associated with advanced heart blocks, needing strict follow‐up by echocardiography, and Holter ECG recording, to promptly recognize and treat pathological clinical expression (Porto et al., 2016). In this case, it represents a syndrome in syndrome, like a *matryoshka doll*.

In 7q35q36.3 syndrome, the *SHH* gene is deleted. The *SHH* gene encodes sonic hedgehog, a secreted protein that is involved in establishing cell fates at several points during the development and his strictly associated with HPE (Echelard et al., 1993; Solomon et al., 2010); nevertheless, the patient had no brain anomalies of HPE spectrum in the MRI scan of brain, but only microcephaly; clinical expression of microform HPE may be represented by midline anomalies as high palate, according to the wide spectrum of clinical manifestations caused by mutation in the *SHH* gene (Kruszka, Hart, Hadley, Muenke, & Habal, 2015; Solomon et al., 2010). Furthermore, the *SHH* gene deletion effect is represented by hypospadias, as a consequence of *SHH* role in genital ectoderm differentiation during urethra genesis (Joodi et al., 2019).

Neurological expression of HPE could include atonic seizures, causing a loss of normal muscle tone, fall down, and prolonged unconsciousness; according to this point, in the absence of clear demonstration of threatening tachyarrhythmias and heart block, we decided to implant a loop recorder for a further clinical follow‐up and better treatment decision (Avari Silva, Bromberg, Emge, Bowman, & Van Hare, 2016).

It is clearly evident that a multidisciplinary approach represents a cornerstone in the daily management of fragile patients affected by insidious genetic disease as 7q35q36.3 deletion.

## 7 CONCLUSION

The deletion of the distal 7q region is a rare chromosomal anomaly associated with multisystemic involvement and considerable cardiac implications, and threatening drug‐related side effect.

A clear phenotypic manifestation has to lead the physicians to a deeper and accurate analysis of the proband genetic constitution, for subsequent finer clinical evaluation of possible gene‐related disease. Physicians and geneticists should get down deep into individual DNA code of syndromic patients, to clearly discover any further possible implication for the correct management, improving many physicians awareness about a widespread cold relief drug that may turn a mother's smile to a screaming nightmare.

## CONFLICT OF INTERESTS

None declared.
